# Effect of Charge State on the Equilibrium and Kinetic Properties of Mechanically Interlocked [5]Rotaxane: A Molecular Dynamics Study

**DOI:** 10.1021/acs.jpcb.2c07645

**Published:** 2023-01-30

**Authors:** Ata Utku Özkan, Dönüş Tuncel, Aykut Erbaş

**Affiliations:** † UNAM-Institute of Materials Science and Nanotechnology, Bilkent University, Ankara06800, Turkey; ‡ Department of Chemistry, Bilkent University, Ankara06800, Turkey

## Abstract

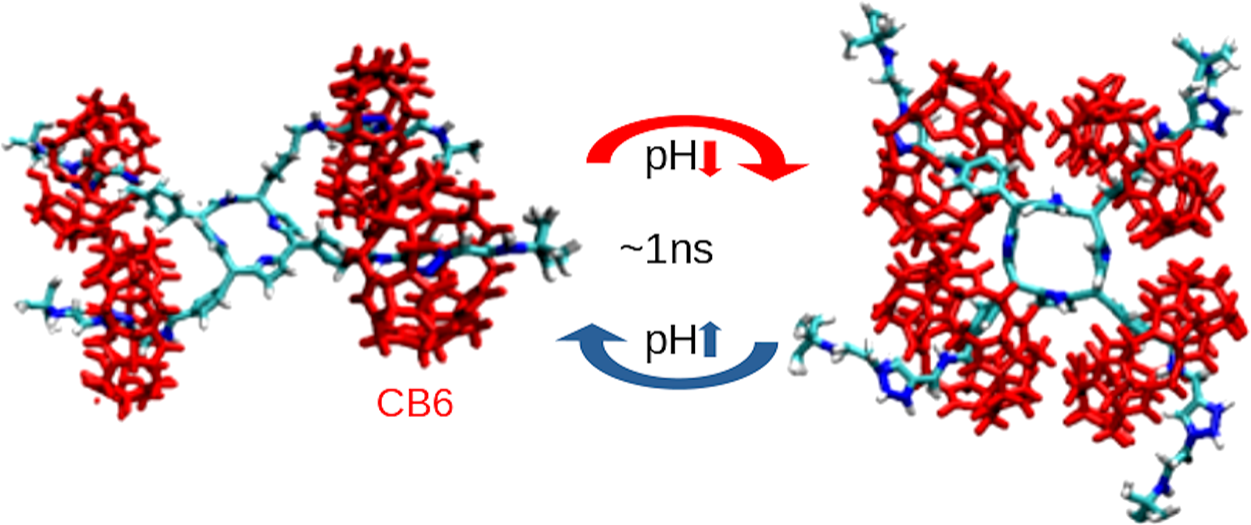

Rotaxanes can exhibit stimuli-responsive behavior by allowing positional fluctuations of their rota groups in response to physiochemical conditions such as the changes in solution pH. However, ionic strength of the solution also affects the molecular conformation by altering the charge state of the entire molecule, coupling the stimuli-responsiveness of rotaxanes with their conformation. A molecular-scale investigation on a model system can allow the decoupling and identification of various effects and can greatly benefit applications of such molecular switches. By using atomistic molecular dynamics simulations, we study equilibrium and kinetics properties of various charge states of the [5]rotaxane, which is a supramolecular moiety with four rotaxanes bonded to a porphyrin core. We model various physiochemical charge states, each of which can be realized at various solution pH levels as well as several exotic charge distributions. By analyzing molecular configurations, hydrogen bonding, and energetics of single molecules in salt-free water and its polyrotaxanated network at the interface of water and chloroform, we demonstrate that charge-neutral and negatively charged molecules often tend to collapse in a way that they can expose their porphyrin core. Contrarily, positively charged moieties tend to take more extended molecular configurations blocking the core. Further, sudden changes in the charge states emulating the pH alterations in solution conditions lead to rapid, sub-10 ns level, changes in the molecular conformation of [5]rotaxane via shuttling motion of CB6 rings along axles. Finally, simulations of 2D [5]rotaxane network structures support our previous findings on a few nanometer-thick film formation at oil–water interfaces. Overall, our results suggest that rotaxane-based structures can exhibit a rich spectrum of molecular configurations and kinetics depending on the ionic strength of the solution.

## Introduction

**Figure 1:**
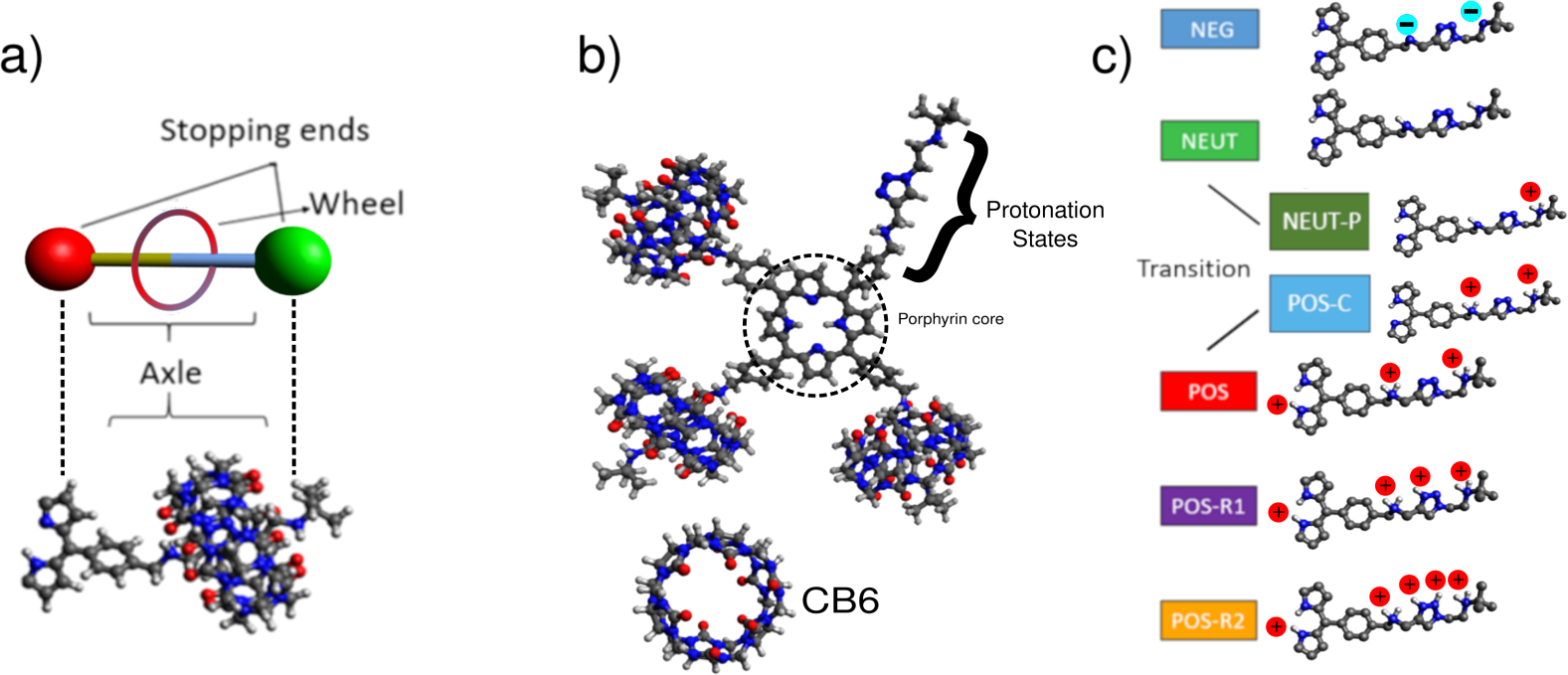
[5]Rotaxane structures used in simulations. (a) Schematics of a single one-axle rotaxane. (b) Molecular structure of [5]rotaxane and cucurbit[6]uril (CB6). For clarity, one of the axles is shown without its CB6 ring. (c) The seven emulated charged states of [5]rotaxane are shown on a single axle with added charges. The pH value decreases starting from the NEUT(pH ∼ 7) to POS-R2 (pH < 4) states. As pH decreases, the outer axle nitrogen is first to protonate (NEUT-P) followed by benzylamine group (POS-C) and finally the central porphyring (POS). Triazole-group nitrogens are protonized only in the POS-R1 and POS-R2 states. The charge states are color-coded throughout this work.

Basic rotaxane structure consists of a rota (e.g., wheel) and an axis component that are held together through noncovalent interactions. The axis component of the structure is terminated by bulky end groups that prevent the wheel slipping off the axis, effectively forming a mechanical bond between the wheel and axle (Figure 1a).^1−4^ External stimuli (i.e., chemical, electrochemical, photophysical) can modify the charge distribution of the rotaxane, which in turn affect the equilibrium position of the wheel on the axle, allowing a translocation of the wheel to a new energy minimum. This mechanism allows rotaxanes to operate as stimuli-responsive molecular switches that can lead to a broad class of novel applications such as cytotoxic kill switches, drug delivery agents, thin films with data storage capabilities, and ultra stable dyes.^5−14^

Higher order rotaxane structures, in which multiple basic rotaxane are incorporated covalently, can exhibit more complex stimuli-responsive behavior.^15−18^ Among them, [5]rotaxane, which is assembled through cucurbit[6]uril(CB6)-catalyzed 1,3-dipolar cycloaddition reaction between tetraalkyne substituted tetraphenyl porphyrin unit and azide functionalized stopping groups, attach four axles together with their corresponding CB6 to the photoactive porphyrin moiety. This architecture with four individually movable CB6 wheels can provide highly stimuli responsive capabilities to the [5]rotaxane that are not present in the CB6-free moieties.^19^ For instance, the experiments showed that [5]rotaxane can function as photosensitive antibacterial drug but this ability vanishes when CB6 were removed from the structure,^20^ suggesting the role of CB6 positional fluctuations in regulating the interactions between [5]rotaxane and bacterial membranes. Furthermore, [5]rotaxanes were shown to covalently self-assemble into thin films in the presence of CB6, indicating the possibility of stimuli-responsive mesoscale structures that can respond physiochemical changes by harvesting the CB6 shuttling along its axle.

Previously, we characterized the pH-responsive behavior of [3]rotaxane (i.e., two CB6 on a single axle) and [5]rotaxene by using ^1^H NMR spectroscopy under various physiochemical conditions.^19,21^ Those measurements revealed that both pH and heat stimuli alter the position of CB6 wheels from one thermodynamically stable recognition site to the other one upon the changes in the protonation states of the axle components. This observation suggests a dynamic shuttling process of CB6 wheels along the axles.^22,23^ However, while ^1^H NMR provides information on the optimum positions of CB6 groups, overall effects of pH changes and of corresponding CB6 movement on the molecular-scale conformation of [5]rotaxane are not clear from such spectroscopic measurement. Moreover, the experiments are limited in quantifying the kinetics of conformational changes of [5]rotaxane resulting from independent shuttling of the CB6 groups under changes in solution pH levels. Indeed, a molecular-resolution examination of pH-dependent behavior of [5]rotaxane, which can focus on the conformational changes and wheel movements separately, can complement the spectroscopic data and further help the development of novel rotaxane-based stimuli-responsive applications.

Molecular dynamics (MD) simulations are proved to be an effective method in understanding the dynamical behavior of macromolecular mechanisms.^24−27^ The [5]rotaxane is sensitive to pH; thus experimentally, its amine groups can be protonated/deprotonated. Effects of different protonation states can be examined by constructing different models of the molecule with corresponding (static) protonation states in MD simulations. Hence, inspired by the previous studies,^14,19^ in this work, we study the equilibrium and time-dependent conformational properties of [5]rotaxane in water and its polyrotaxanated network structures at water-chloroform interfaces by considering various physiochemical as well as exotic charged states of [5]rotaxane. Overall, our simulations show that the protonation state of [5]rotaxane determines molecular configuration as well as kinetics of the shuttling motion of CB6 groups. Furthermore, beyond the single-molecular level analysis of [5]rotaxane, we also simulate pH-responsive conformational changes of polyrotaxanated network versions of [5]rotaxane that can be synthesized through interfacial polymerization of tetraalkyne and tetraazide functionalized porphyrin in the presence of CB6 at the water–chloroform interface. Simulations show that these network structures are highly stable at liquid–liquid interfaces and could exhibit pH-dependent-porosity.

## Method

### All-Atom Simulations

#### Single [5]Rotaxane

The molecular structure of the [5]rotaxane molecule is first constructed in a PDB format via Chem3D software.^28^ Bonded interactions of [5]rotaxane are parametrized from the GROMOS96 54a7 force field parameter set.^29,30^ CB6 ring charge topology is generated from AM1 optimized geometry and MOPAC charges by using the Automated Topology Builder (ATB).^31,32^ Each [5]rotaxane molecule is solvated in SPC/E water by using the GROMACS molecule insertion algorithm together with its neutralizing (positive or negative) counterions such that in each simulation box the net charge is zero. SPC/E water model is chosen due to its better performance in capturing the water dynamics and structure as well as its relative precision in hydrogen-bonding lifetimes.^33,34^ The force-field dependency is further investigated by simulating the nonexotic [5]rotaxane protonation states with the general AMBER force field (GAFF) and with RESP fitting HF/6-31* level optimized electrostatic potentials.^35−39^ No qualitative difference is observed (Figure S1) as we will discuss further in the text.

Each simulation box containing a single molecule, ions, and water molecules is energy-minimized with the steepest descent algorithm. After the minimization, each system is run for 50 ns with an integration time step of 2 fs for additional relaxation under a constant pressure of 1 atm at a constant temperature of 300 K. The stability of each configuration is ensured by extending each simulation for additional 30 ns under the same conditions. The Parienello Rahman pressure coupling and the V-rescale (Berendsen) temperature coupling are used to maintain thermodynamic conditions throughout the simulations.^40−42^ The Verlet cutoff scheme is used with a nearest neighbor search and with a 1 nm cutoff for all nonbonded interactions.^43^ For all bulk simulations, the dimensions of the cubic simulation box are 11 × 11 × 11 nm^3^. All simulations are run with the GROMACS simulation package with periodic boundary conditions, and built-in GROMACS algorithms are used for analysis.^44−54^

#### Polyrotaxanated Network

The polyrotaxanated network structures are constructed by bonding four [5]rotaxane molecules by their terminal ends. Bonded interaction and charge parametrization of polyrotaxane is similar to the single-molecule case. To form the network, the terminal parts of the axle including the triazole group are removed, and two rotaxane molecules are connected with an added triazole group (Figure S2). This PN structure is identical with the one reported in ref (14). For the interactions between chloroform and the rest of the system, the ATB Database is used. The resulting supramolecular structure is placed at a water–chloroform interface and their free ends are bonded to the ends of periodic images of the initial PN in the neighboring simulation boxes. The water–chloroform interface is prepared by first equilibrating water and chloroform phases individually by inserting packing number of water and chloroform molecules into the 11 × 11 × 7.5 nm^3^-sized boxes. Following, an energy minimization and constant-volume equilibration for 2 ns are performed for each phase. Then, equilibrated water and chloroform molecules are placed in the bottom and top half of the simulation box, respectively, sandwiching the periodic network structure. Initially, the total simulation box dimensions are set to 11 × 11 × 18 nm^3^ for the interface simulations. All polyrotaxane simulations are run for 50 ns at 1 atm pressure by using the Parienello Rahman semi-isotropic pressure-coupling method at a temperature of 300 K.

#### Analyses

In order to analyze the properties of the [5]rotaxane structures obtained at the end of the simulations, several computational analysis tools are employed. As a measure of overall spread of the molecule, the radius of gyration (*R*_g_) is calculated by
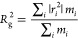
where *m*_*i*_ is the mass of atom *i*, and *r*_*i*_ is the position of atom *i* with respect to the center of the mass of the molecule. The solvent accessible surface area (SASA) are calculated with 0.14 nm probe radius and averaged over time.^55,56^ For calculation of the hydrogen bonding, a commonly adapted geometrical definition is used.^57^ For all visualization purposes the Visual Molecular Dynamics (VMD) program is used.^58−65^

### Charge Distribution

In order to simulate various protonation states of [5]rotaxane, seven different charge configurations are obtained by modifying the partial charges on nitrogen atoms on the axle and core parts of the molecule while the electrons of axle carbon hydrogens are neglected^66,67^ (Figure 1b). No charges on carbon atoms that can arise due to conformational changes of [5]rotaxane are considered. Nitrogen–hydrogen pairs are assigned partial charges borrowed from the amine and aromatic groups defined in GROMOS96 54a7 force field. To further crosscheck the assigned charges of donor nitrogen atoms, the electronegativity equalization method is used to compute and compare the charge of individual atoms in the porphyrin.^67^ Specifically, the NEUT state is constructed with partial charges assigned only on amine groups. The POS state is achieved via adding a positively charged hydrogen (i.e., +1.0*e*, where *e* is the elementary unit charge) to the neutral nitrogen sites. The same procedure is repeated on triazole nitrogen to obtain POS-R1 and POS-R2 states (Figure 1b). For the NEG state, the hydrogen-free nitrogen atoms are assigned negative charges. Sodium and chloride ions are used as counterions to neutralize the molecular charges for appropriate charge states. The partial charges of nitrogen and hydrogen are given in Table 1. Note that among these seven charge states, NEUT and POS are experimentally realizable at a pH range of 4–7, whereas the states NEG, POS-R1, and POS-R2 are exotic states that are defined to simulate the limiting regimes.

**Table 1:** Partial Charges Assigned to Individual Nitrogen and Hydrogen Atoms to Obtain Various Change States of [5]Rotaxane, also Schematically Shown in Figure 1^a^

	NEG	NEUT	POS	POS-R1	POS-R2
N	–0.9225	–0.31	–0.31	–0.31	–0.31
NL	–0.31	–0.31	–0.31	–0.31	–0.31
NT	0.0	0.0	0.0	0.0	0.0
HN	N/A	0.31	0.31	0.31	0.31
HP	N/A	N/A	1.0	1.0	1.0
HT	N/A	N/A	N/A	1.0	1.0

a N refers amine group nitrogen on the axle parts, NL refers to nitrogen on center porphyrin core, NT refers to nitrogen on triazole groups, HN refers to hydrogen of N, HP refers to hydrogen added to nitrogen for protonation, and HT refers to hydrogen of NT.

## Results and Discussion

### Charge-Dependent Conformation of Single [5]Rotaxane in Salt-free Water

**Figure 2:**
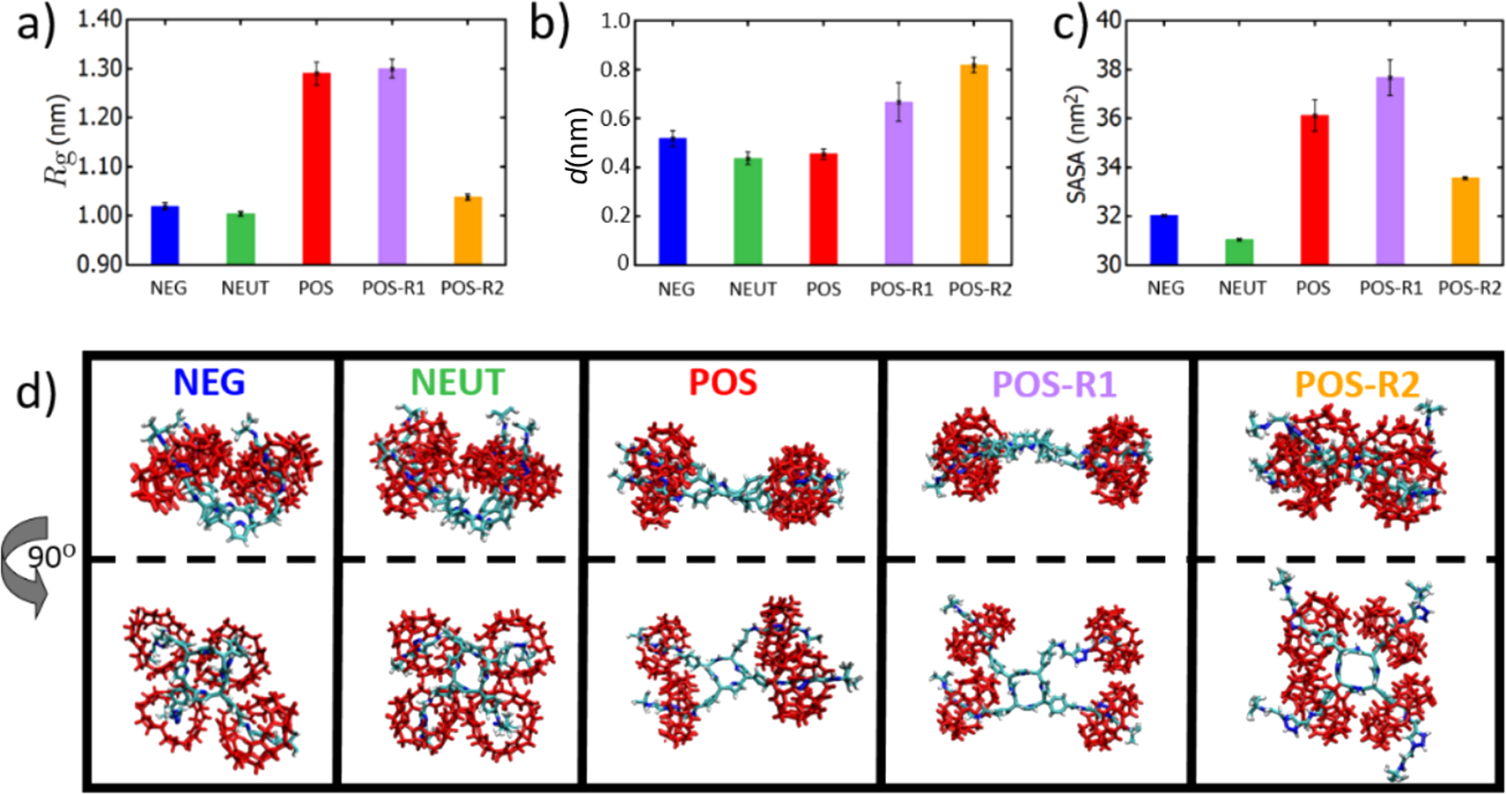
Effect of charge state of [5]rotaxane on (a) radius of gyration, (b) the average distance between triazole groups and corresponding (identical axle) CB6, and (c) the solvent accessible surface area. (d) Representative simulation snapshots of equilibrium configurations of [5]rotaxane for various charge states at the end of ∼50 ns long simulations. For clarity, water molecules are not shown, and the CB6 groups are colored red.

In our MD simulations, single [5]rotaxane molecules with various charge-states are simulated in explicit water, in which individual water molecules and counterions can interact with the molecule via steric and electrostatic interactions. We quantify configurational properties of [5]rotaxane structures for each charge state by analyzing equilibrium structures upon at least 50-ns-long simulations. Specifically, we analyze solvent accessible surface area, radius of gyration (i.e., *R*_g_), nonbonded interaction energies, and hydrogen bonding.^55,56^ We consider five main charge states of [5]rotaxane as described in Figure 1b and refer to these states as follows: a negatively charged (i.e., NEG), neutral (i.e., NEUT), positively charged (i.e., POS), second positively charged but with additional charges on triazole groups (i.e., POS-R1), and finally with fully protonated triazole groups (i.e., POS-R2). The transition in between NEUT and POS states gives rise to two additional intermediate states (Figure 1b). We refer to these states as NEUT-P and POS-C, which are also examined thoroughly. Throughout each simulation, the charge state of the molecule does not change, and each charge of [5]rotaxane is neutralized by its corresponding solution-phase counterion. We initiate all simulations by using the same initial configuration. Our simulations in general indicate highly charge dependent molecular configurations, albeit with weakly distinguishable molecular dimensions (Figure 2a).

At first look, the radii of gyration (*R*_g_) of [5]rotaxane do not exhibit a significant difference with variations in the molecule’s charge state except for POS and POS-R1 states: the *R*_g_ values lie around *R*_g_ ∼ 1 nm for NEG, NEUT, and POS-R2 states (Figure 2a). The POS and POS-R1 states show slightly higher *R*_g_ values than other charge-states. Notably, despite the drastic charge difference between POS-R2, and NEG and NEUT, *R*_g_ values are similar within the error bars, suggesting that molecular dimension could be a weak descriptor to quantify the charge states (Figure 2a).

A visual inspection of our representative equilibrium-simulation snapshots reveals that molecular configurations are vastly different for each charge state despite relatively close *R*_g_ values (Figure 2c). Relative orientation of porphyrin arms (porphyrin core + axle parts) are highly charge dependent; in neutral (i.e., NEUT) and negatively charged (i.e., NEG) states, the four CB6 rings are positioned in a way that the porphyrin core is highly exposed to water molecules but in an asymmetric fashion (Figure 2d). This configuration is achieved by bringing CB6 rings closer to each other while maintaining their average positions near the corresponding triazole group exposing the core to water (Figure 2d). Contrarily, for the POS and POS-R1 states, CB6 groups are more solvated, resulting in an open [5]rotaxane conformation with relatively larger *R*_g_ values (Figure 2a, d). Interestingly, in the POS-R1 state, CB6 rings interact with each other in the groups of two in a way that only two of CB6 are close to the terminal of the axle (Figure 2d). The most dissimilar configuration occurs for the POS-R2 state; in this state, CB6 rings are positioned near the core of the [5]rotaxane, effectively reducing *R*_g_ with a distinctly different mechanism than those occurred for the NEG and NEUT states, in which the core is exposed asymmetrically (Figure 2d). We conclude that the inward position of the CB6 rings reduces the molecular size of [5]rotaxane (Figure 2a).

Given that average positions of CB6 rings could be a major determinant of the overall molecular configuration in each charge state, we calculate the average optimum positions of the rings in our equilibrium configurations. In most cases, the stable average position for CB6 rings appears to be on 1,2,3-triazole groups since the nitrogen on triazole rings do not have a net charge except at the state POS-R1 and POS-R2 cases (Figure 1c). This can be also seen in Figure 2b, in which the average distances between the centers of mass of triazole and CB6 rings for each axle are shown for the five charge states. For all cases except POS-R1 and POS-R2, triazole and CB6 groups are in close physical proximity, consistent with the equilibrium configurations in Figure 2b. Thus, we conclude that charge state does not effect the location of CB6 rings if triazole groups are not protonated. Notably, CB6 rings are repelled from both positively or negatively charged terminals in a similar fashion, suggesting the role of steric interactions keeping [5]rotaxane molecule intact while allowing a positional flexibility to its rings.

In our previous experimental studies,^19^ as the solution pH was increased gradually (from 3.5 to >7), the H NMR spectra of CB6’s protons exhibited a broadening, suggesting multiple stable locations along the corresponding axis. In the same experiment, the triazole-group proton exhibited a spectroscopic signal change due to CB6 dethreading. In the simulations, such an increase in pH corresponds to POS → NEUT transition, for which the triazole-CB6 distance seems to be weakly affected (Figure 2b,d). Further, while the previous H NMR data indicates an exposed triazole group in the NEUT state, we rather observe triazole groups blocked by CB6 wheels in the collapsed [5]rotaxane configurations (Figure 2d). Notably, in our test simulations with the GAFF force field, we observe the same CB6 positions at the NEUT state (Figure S1), suggesting an alternative mechanism that would cause a ^1^H NMR signal originating from the exposed triazole groups such as oligometrization of multiple [5]rotaxanes.

**Figure 3:**
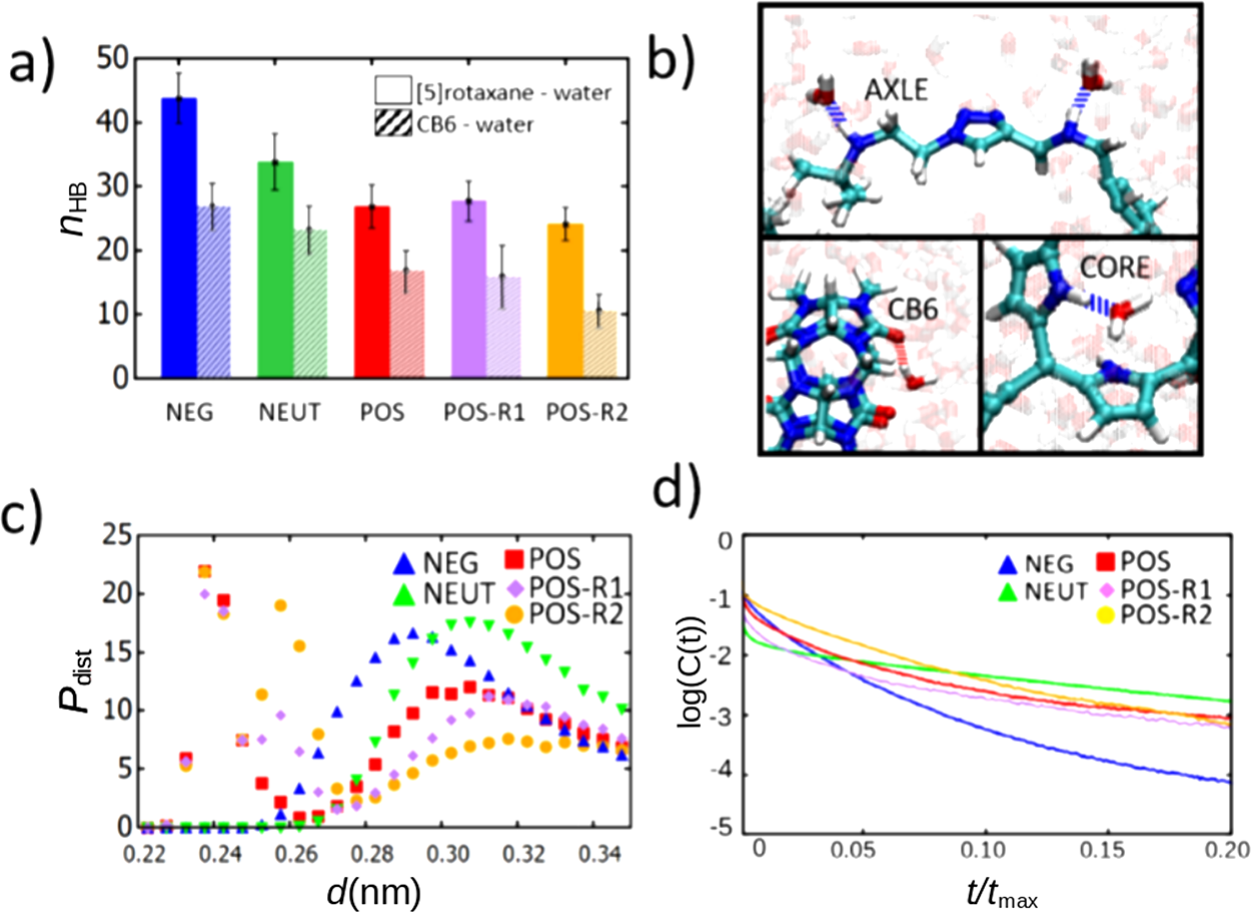
(a) Hydrogen bonding between water and [5]rotaxane at various charge-states calculated via time-averaging of MD simulation trajectories. (b) Representative snapshots showing the hydrogen-bonding groups of the [5]rotaxane. The dashed blue and red lines refer to NH–O and OH–O types of hydrogen bonds, respectively. The water molecules in the background are shown transparent for clarity. (c) The distribution of hydrogen bond lengths various charge states. (d) Hydrogen bond lifetime auto correlation function normalized over time.

To gain further insight on the molecular interactions leading to various [5]rotaxane conformations, we next quantify the molecular configurations by calculating the solvent accessible surface area (SASA) and hydrogen bonding between solvent and the molecules for each charge state (Figure 2c, Figure 3a). A higher SASA value is an indication of stronger interactions with solvent molecules.^55^ Consistent with the visual analyses, the protonation of triazole greatly increases the SASA by placing the CB6 rings away from the core porphyrin. In our charge-neutral and negatively charged [5]rotaxanes, the SASA values are the smallest due to the folding of the axle parts and increasing interactions in between the four CB6 rings. Interestingly, the POS-R2 state blocks the core from water via CB6 groups, resulting in a relatively smaller SASA value even though the individual axles are of a stretched configuration (Figure 2c-d). Notably, the conformational variations of [5]rotaxane at different charge states distinctly manifest themselves at SASA profiles, suggesting that SASA could be used as a metric to characterize configurational kinetics as we will discuss later.

Hydrogen bonding between the solvent and components of [5]rotaxane also exhibit a strong dependence on the charge state of [5]rotaxane. The general trend is that as the protonation of the [5]rotaxane increases, the total number of hydrogen bonds *n*_HB_ with solvent decreases (Figure 3a). The maximum number of hydrogen bonds is observed for the NEG state with *n*_HB_ ≈ 44, while the minimum is *n*_HB_ ≈ 24 for the state POS-R2. This indicates that the NEG state is relatively more water-soluble than other charge states due to hydrogen bonds that it can form with water via its nitrogen atoms. In other charge states, amine or amino groups are the main source for hydrogen bonding (Figure 1b). The contribution of CB6 rings to the total hydrogen bonding also decreases as protonation is increased (Figure 3a). The hydrogen-bonding contributions due to the porphyrin core and axles do not show a significant change for various charge states and is around *n*_HB_ ≈ 10 within the error bars (Figure 3b). This suggests that the position of CB6 groups indeed is a determinant of the solvation and self-assembly properties by either controlling the hydrophilic interactions or the blocking of the core sterically. For instance, the arrangement of CB6 rings near the core in the POS-R2 state can sterically block the porphyrin core from establishing hydrophobic interactions but also limits the hydrogen bonding capacity of [5]rotaxane. This situation is opposite in the negatively charged and neutral [5]rotaxane moieties; the close arrangement of CB6 wheels can expose the core and increases the hydrophilicity of [5]rotaxane simultaneously.

When we analyze the distance distributions of hydrogen bonds (i.e., average donor–acceptor atom distances), we observe that protonated nitrogen atoms of positively charged states have shorter hydrogen bonds on average as compared to the negatively charged and charged neutral states as shown in Figure 3c. Further, the NEG state has shorter living hydrogen bonds as can be inferred from the more rapid decay of the hydrogen-bond autocorrelation function (Figure 3d). Overall, our simulations demonstrate that charge state and the equilibrium location of CB6 rings greatly affect the solvation properties of [5]rotaxane via alterations in SASA and hydrogen bonding.

### Effect of Charged Porphyrin Core on the Structure

**Figure 4:**
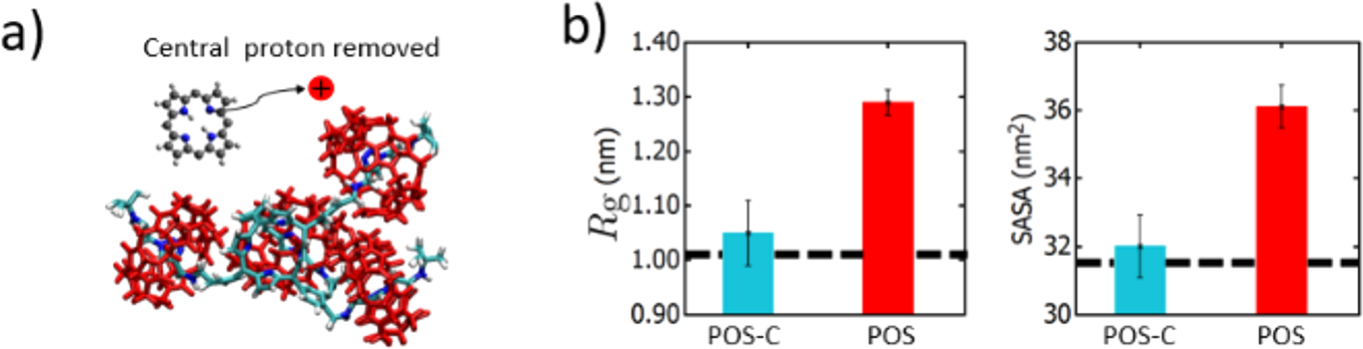
(a) Representative equilibrium conformation of [5]rotaxane when its central proton is removed from the POS state. (b) Comparison of *R*_g_ and SASA values of POS states with and without its and core charge removed. The dashed lines denote the averages of *R*_*g*_ for NEUT and NEG states.

Our simulations suggest that the charge of porphyrin core can contribute to distinct charge-dependent solubility properties of [5]rotaxane (Figure 1c, Figure 2d). Hence, we run a series of test simulations by deleting the positive charge on the porphyrin of the [5]rotaxane POS state (Figure 4a). We refer to this state as the POS-C state. We should note that this charge state is chemically improbable. However, it can serve as a limiting regime to demonstrate the drastic effects of minor chemical modifications on rotaxane structures.

The equilibrium dimensions and solutions properties of [5]rotaxane with a protonated core is somewhat similar to the collapsed configurations that we observe for the NEG and NEUT states with no core charges (Figure 4). Notably, positional fluctuations of the axle parts are more drastic as compared to those observed for the POS state, as manifested by higher standard errors of *R*_g_ and SASA values (Figure 4b). A configurational distinction of the POS-C state from other positively charged states (i.e., POS, POS-R1, POS-R2) is the interaction among CB6 rings; while the NEG and NEUT states have all CB6 groups closely packed (Figure 2d), for the POS-C state, at a given time, only the second-nearest neighboring CB6 groups interact closely while the other two prefer an extended configuration, resulting in an asymmetrical molecular configuration (Figure 4a). This configuration is also different than those observed for NEUT and NEG states, in which CB6 rings join away from the poryprin core, exposing the core to the solvent molecules (Figure 2d). When we increase the statistics by running 10 more replicas with different initial velocity distributions, this asymmetrical configuration persists, albeit with different CB6 pairings (i.e., either CB6(1) and CB6(3) or CB6(2) and CB6(4) pairs interact closely as illustrated in Figure 4a). Overall, these simulations demonstrate that the core protonation stabilizes an extended molecular configuration and prevents a folded [5]rotaxane conformation.

### Kinetics of Conformational Changes of [5]Rotaxane

In the previous sections, we show that average position of CB6 rings and resulting molecular conformations of [5]rotaxane are highly dependent on the charge state (e.g., the ionic strength of solvent). We next ask the question how fast the [5]rotaxane can switch from one conformation to another upon a stimulus that can alter the charge state of the molecule (e.g., by changing the solution’s ionic strength). In order to achieve this, we assign the equilibrium conformation of a charge state as the initial configuration of simulations but with a new charge state (e.g., use NEUT equilibrium configuration to run POS simulations, etc.). In this way, we observe the effects of rapid ionic strength variations of solution on the time-dependent transitions of the molecular configurations.

Considering five main charge states given in Figure 1c, there are 2 × 9 = 18 possible transitions including both forward (e.g., NEUT → POS) and reverse (e.g., POS → NEG) transitions. However, the transitions between the states with similar molecular conformations (e.g., from NEG to NEUT) do not show any significant time-dependent conformational changes when SASA and *R*_g_ values are monitored (Figure 2d, Figure S3 and S4). The transitions being initiated from or to NEUT/NEG are also not observable due to the blockage of CB6 groups by the initial collapsed state of the four axles at the beginning of a simulation (Figure 2d).

**Figure 5:**
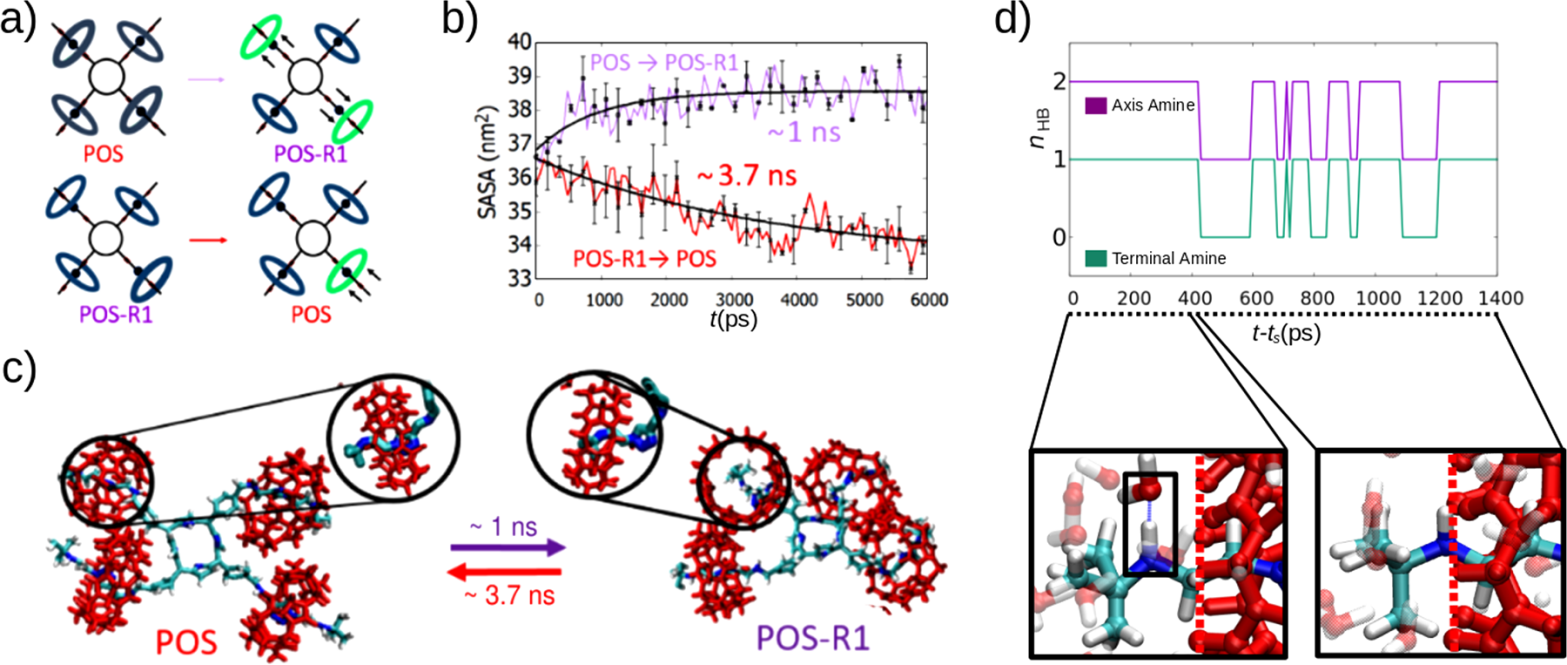
Kinetic analyses of the transitions between the POS and POS-R1 states. (a) Illustration of CB6 movements during the transitions. The green rings denote CB6 movements. (b) Solvent accessible surface area versus time for the transition (POS ↔ POS-R1). The curved are fit functions *f*(*t*) = 1 ± exp(−*t*/τ_SASA_), where τ_SASA_ is the characteristic time of the transition. (c) Representative simulation snapshots of the shuttling motion of CB6 groups toward/away outer terminal amine. (d) The time traces of the number of hydrogen bonds between water and amine groups of a single [5]rotaxane axle for the POS-to-POS-R1 transition. The dashed vertical lines in the bottom panel show the edge position of the CB6.

We observe the most drastic changes in the conformational and solution properties of [5]rotaxane in the transitions, in which the POS state is the final state and *vice versa* (Figure 5, Figure S5 and S6). Almost in all of those transitions, time-dependent configurational transitions were accompanied by simultaneous shuttling motion of at least one CB6 ring along its axle (Figure 5a-c). For instance, in the forward transition (i.e., POS → POS-R1), the shuttling response of CB6 rings are not all-in-once, while two second nearest neighboring CB6 groups move toward terminal amine groups of the axle parts, the other two maintain their positions (Figure 5a). In reverse transition (i.e., POS-R1 → POS), only one CB6 moves toward to its native position near triazole group, and no complete conformational reversal is observed, suggesting the need for additional stimuli for this transition to take place (Figure 5a).

The shuttling of CB6 rings along the axles can also manifest itself as apparent alterations in inter- and intramolecular interactions. Hence, we quantify time-scales of conformational changes of [5]rotaxane by monitoring time traces of hydrogen bonding and SASA profiles. Averaging over 20 simulation replicas reveal that SASA responds rapidly upon the alteration of the charge state before reaching a saturation value (Figure 5b), whereas the total hydrogen bond population between [5]rotaxane and water *n*_HB_ responds rather weakly (5%) (Figure S7). However, isolating the time dependent hydrogen bonding analysis to one shuttling axle reveals that CB6 moving to the terminal groups sterically disrupts the hydrogen bonding between the amine groups and water (Figure 5d). Due to the positional fluctuations of CB6 about the triazole, the average number of hydrogen bonds that the axle can form with water drops to *n*_HB_ ≈ 1.5 from *n*_HB_ ≈ 3, suggesting an energy barrier of around two hydrogen bonds between the two states.

Given that SASA values are a better descriptor of molecular configurational changes than *R*_g_ and *n*_HB_, we obtain characteristic time scales of transitions by fitting the SASA data to simple exponential functions. We obtain a forward transition times of around τ_SASA_ = 935 ± 10 ps ≈ 1 ns via a fit function in the form of *f*(*t*) = 1–exp(−*t*/τ_SASA_) for the POS → POS-R1 transition. In the reverse transition (i.e., POS-R1 ↔ POS), the SASA decreases even below the SASA value of the state POS obtained from the equilibrium simulations (Figure 2c). Fitting the function *g*(*t*) = 1 + exp(−*t*/τ_SASA_) to the SASA data leads to a reverse transition time of around τ_SASA_ = 3698 ± 20 ps ≈ 3.7 ns, which is slower than that of the forward transition. In general, we obtain transition times on the order of several nanoseconds for other transitions as well (Figures S4–S8). If assume that the entire transition of [5]rotaxane can be described as a single-barrier crossing process, transition time could be written as τ_SASA_ ≈ τ_0_ exp(*U*/*k*_B_*T*),^68^ where τ_0_ ≈ *a*^3^/*ηk*_B_*T* is the transition time in the absence of any energy barrier, *a* is the characteristic length scale of the transition, η is the solvent viscosity, *k*_B_ is the Boltzmann constant, and T is the absoulete temperature. Assuming τ_0_ ≈ 0.25 ns for a particle of size *a* ≈ *R*_*g*_ ≈ 1 nm in water (η = 10^–3^ Pas) gives τ_SASA_ ≈ 1 ns with *U* ≈ 1*k*_B_*T* for the POS → POS-R1 transition after taking 1*k*_B_*T* ≈ 4 pN × nm (at T = 300K). For the POS-R1 → POS transition, an energy barrier height of *U* ≈ 3*k*_B_*T* is required to obtain τ_SASA_ ≈ 4 ns. Indeed, *U* ≈ 3*k*_B_*T* is the energy of 1–2 hydrogen bonds,^69^ consistent with the hydrogen-bond data that we discuss above (Figure 5d).

**Figure 6:**
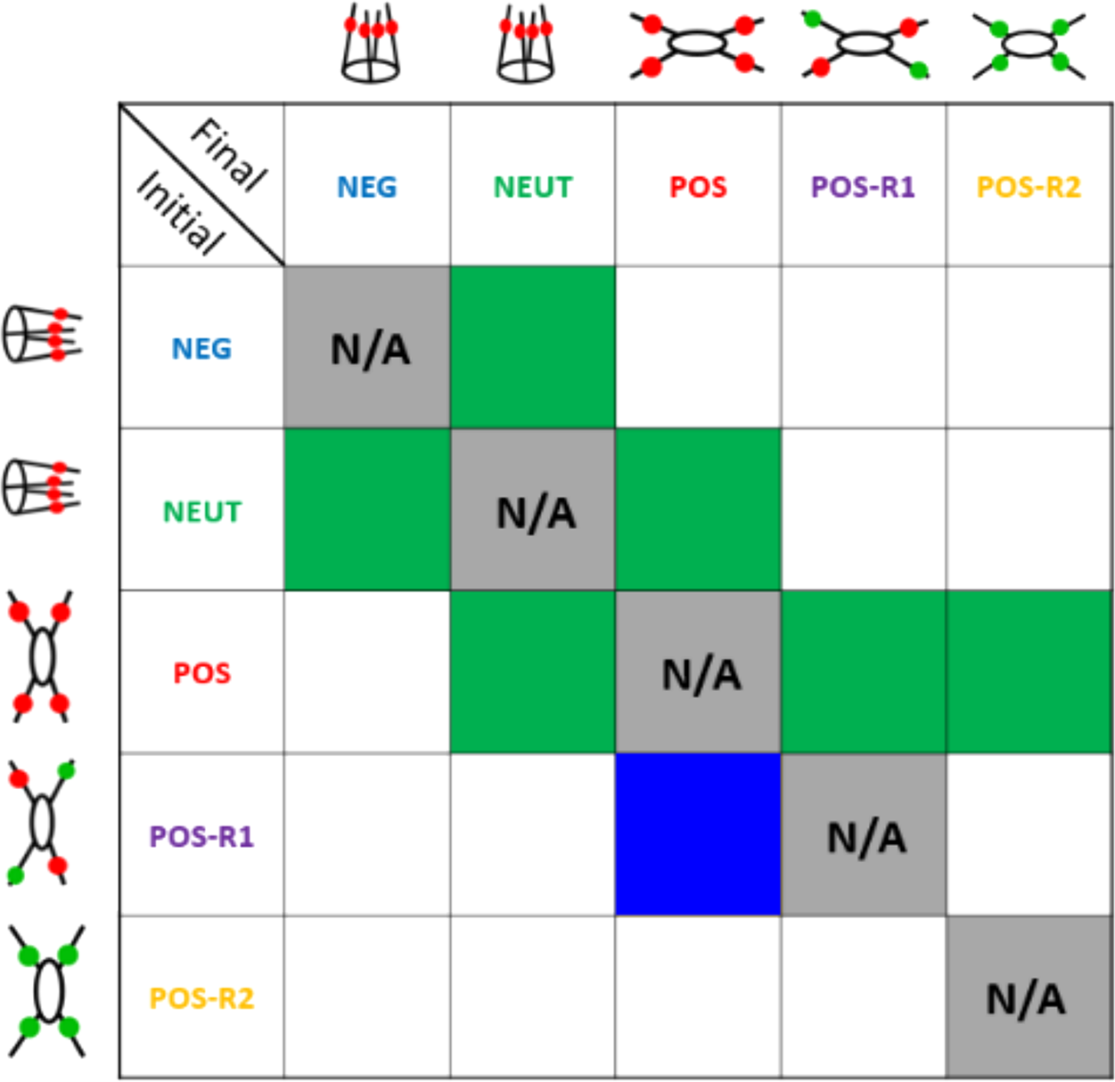
Transition matrix between multiple states. Green cells denote complete transitions from an equilibrium initial state to a final equilibrium state. Blue square denotes partial transition, in which not all but some CB6 rings can shuffle to their equilibrium positions. In schematics of [5]rotaxanes, green and red beads refer to CB6 rings leaving or staying at their corresponding trizaole group, respectively.

Consistently, a visual inspection of transition simulations reveal that the reason behind the time-asymmetry between forward and reverse transitions appears to be a slower and partial relocation of CB6 rings along the corresponding axles (Figure 5a,c). Among the 20 replicas of POS-R1 to POS simulations, some (i.e., 5 in 20) singular CB6 ring does relocate back onto the triazole group, resulting in a partially stabilized POS state, which in turn explains the decrease of SASA value below the equilibrium POS state; as the CB6 located on the tip of the axle bends the axle toward core porphyrin, the SASA lowers further. This suggests that while protonization on its own is enough of a factor for forward shuttling, additional interference such as heat is required to restore the system completely to its initial POS state. Overall, our simulations suggest that [5]rotaxane can respond to changes in the charge states quite rapidly by allowing shuttling of CB6 rings along the axles while demonstrating a one-way controllable switch property. We summarize the simulation results in a transition matrix given in Figure 6.

### The Role of Axle Conformation on the Shuttling Kinetics

**Figure 7:**
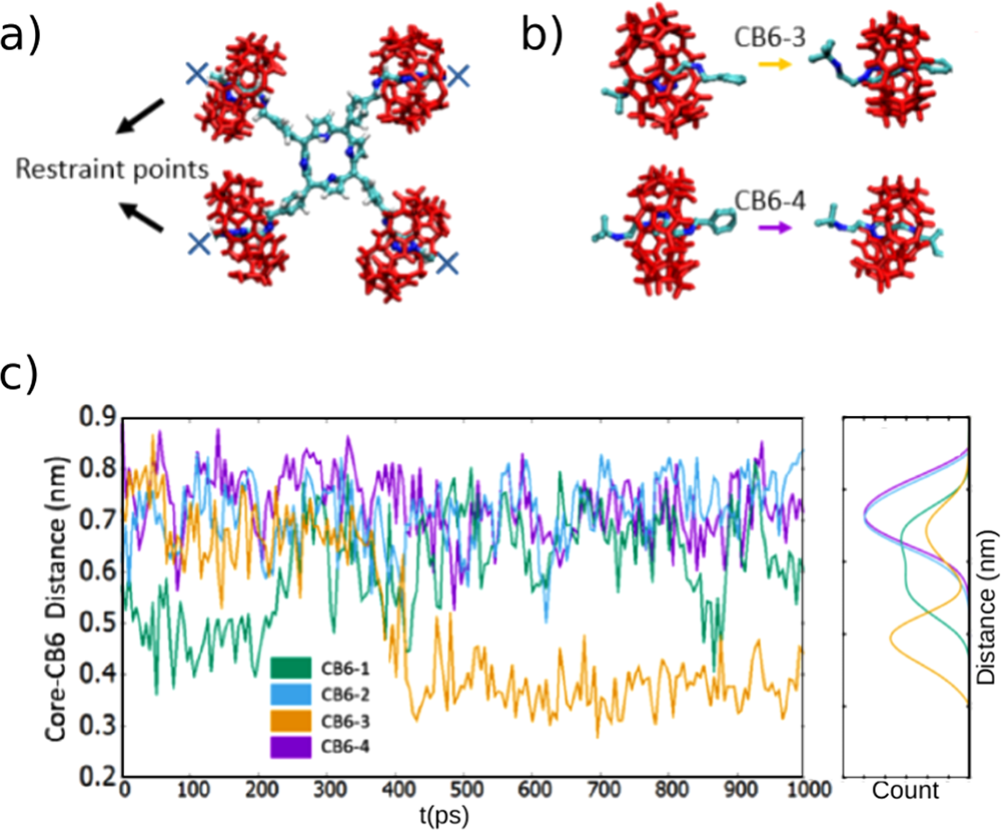
Effect of axle conformation on CB6 shuttling. (a) Restrained NEUT-P state model that has a spring-like potential applied to the axle end points. (b) Representative snapshots of two selected CB6 groups moving along their axles. (c) The asynchronous and subtle shuttling of different CB6 toward and away from the porphyrin core are indicated by the time traces of minimum distances between CB6 and core nitrogen.

In our transition simulations initiated from NEUT/NEG states, the collapsed axle parts of the molecule do not allow any CB6 movement or any large-scale change in the conformation of [5]rotaxane (Figure 2d). Thus, we question the role of axle conformation on the overall conformational changes of the molecule. In order to isolate the CB6 movement from the axial collapsing, we employ spatial constraints to the terminal carbon atoms of [5]rotaxane to keep the axles stretched and spread (Figure 7b,c). We decide to use the semiprotonated state (NEUT-P), which is between NEUT ↔ POS states as the benzylamine nitrogen are first to acquire positive charge with decreasing pH. Notably, this state is recently experimentally realized by Tuncel et al.^19^

When axles are kept stretched by the constraints, the CB6 groups are mobilized along the axle (Figure 7b,c). Each CB6 moves independently from the others along the axis; CB6 groups fluctuate along the axles, toward and away from the benzylamine groups (Figure 7c). Within the simulation time, we observe binomial distributions for the core-CB6 distances, suggesting more than one thermodynamically stable positions for the corresponding wheel. Such dynamic positional fluctuations of CB6 along the axle, between multiple sites, with increasing pH was also observed in the H NMR spectra by monitoring the intensity of triazole proton signals.^19,21^ Overall, these model simulations suggest that axle conformation can impact the conformational response of [5]rotaxane to external stimuli.

### Poly-[5]rotaxane Network at Hydrophobic–Hydrophilic Interfaces

**Figure 8:**
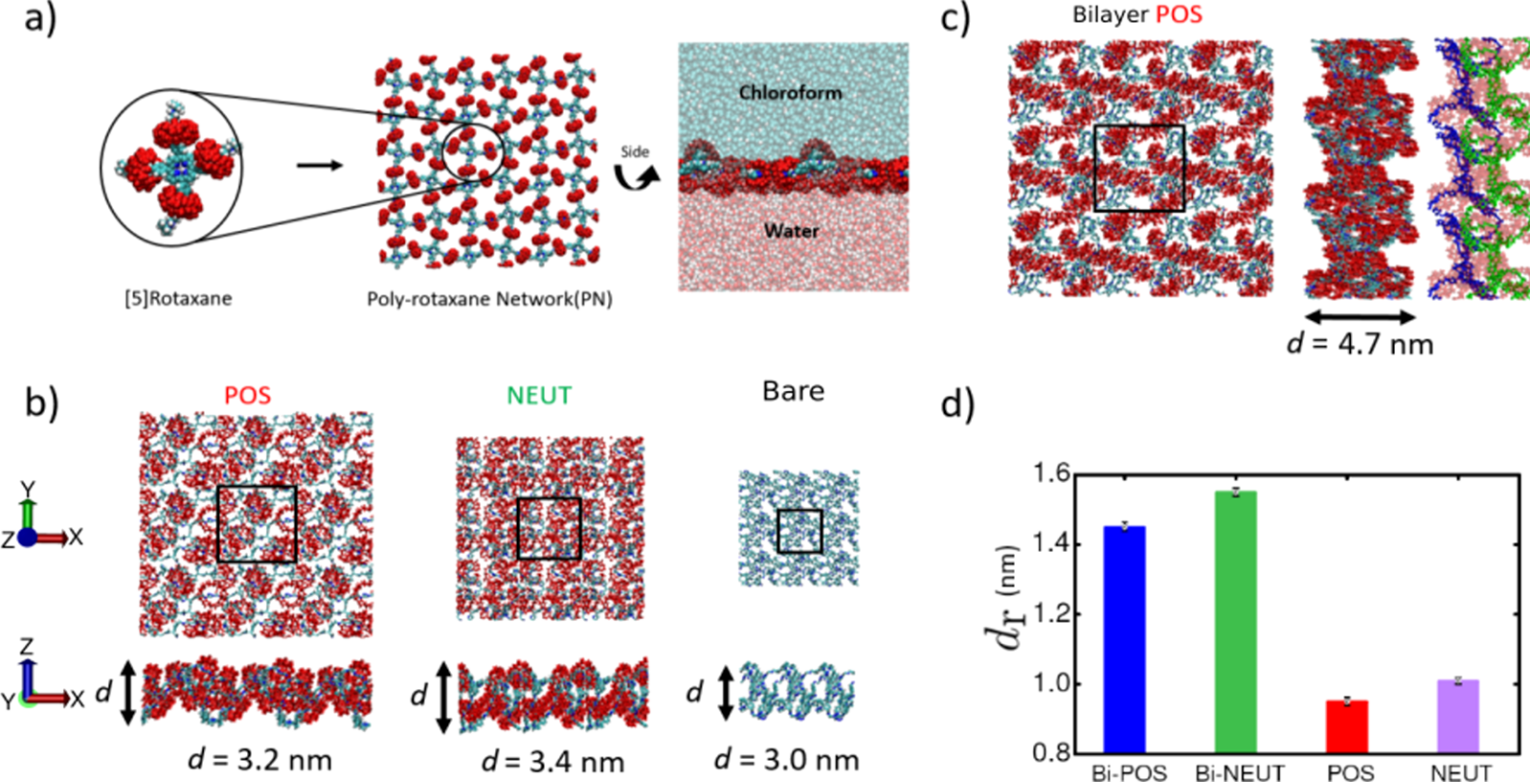
Effect of charge state on polyrotaxane network: (a) Construction of polyrotaxane-network structures from a single [5]rotaxane via periodic bonds. The structure is placed at the interface between water and chloform. (b) Representative simulation snapshots of various polyrotaxane-network structures after 50-ns-long simulations for various charge-states. The bare refers to the cases, in which CB6 are removed prior to running the simulation. (c) Bilayer polyrotaxane structure. On the left side, the same structure are shown by coloring the porphyrin of each layer differently. (d) Calculated porosity values for various polyrotaxane-network structures.

Recently, the polyrotaxanated 2D-network version of [5]rotaxane was reported.^14^ The network was synthesized through interfacial polymerization of tetraalkyne and tetraazide functionalized porphyrin in the presence of CB6 at the water–chloroform interface. We also investigate pH-responsive conformational changes of these structures at the water–chloroform interface in accord with the experimental conditions.^14^ Polyrotaxane structures are constructed by introducing covalent bonds between the termimal groups of [5]rotaxane molecules to the terminal groups of [5]rotaxanes in the 4 neighboring periodic boxes in *x* and *y* directions (see Methods section and Figure 8a).

Our simulations in general show that polyrotaxane macromolecular 2D networks can maintain their configuration stably at the interface owing to the highly amphiphilic nature of [5]rotaxane regardless of charge-state of the constituting [5]rotaxane. Visual inspection reveals that the porphyrin groups are closer to chloroform phase while CB6 rings are mostly immersed in water phase (Figure 8a). At the interface, a well-preserved planar structure, in which CB6 rings form a quasi-lattice, persists throughout the duration of our 20-ns-long simulations. Notably, a single nonperiodic rotaxane molecule immersed in water or air–water interface is settled on a collapsed state (Figure S9), suggesting the requirement of a large scale assembly of polyrotaxanes at the interface to obtain such planar 2D networks.

The distance between the CB6 groups on the surface of the polyrotaxane structure exhibits a charge-state dependence. As the charge of the [5]rotaxane is increased, the network structure expands in lateral directions, a property reminiscent of polyelectrolyte hydrogels.^70^ Note that in simulations, the surface area of the network is determined by the lateral pressure, which is set to 1 bar. Due to the lateral expansion, the polyrotaxane network composed of [5]rotaxanes with NEUT states leads to a denser CB6 arrangement on the surface as compared to the networks composed of positively charged [5]rotaxanes, in accord with the previous experiments.^14^ Note that the arrangement of CB6 groups does not seem to change the thickness of the film, which is around ∼3.0 nm (Figure 8b). In a subset of simulations, we remove all CB6 rings from the network structure to investigate steric effects of rings on the network structures. These simulations lead to a highly collapsed 2D network (Figure 8b, right panel), suggesting the role of CB6 rings in the stability of an extended 2D network configuration.

Previous experiments also suggested the multilayer formation of polyrotaxane network, in which more than two polyrotaxane networks stack on the top of each other. When we set up two network layers in a stacking configuration, we observe a highly stable multilayer structure (Figure 8c), in which CB6 closely packed and individual layers stay adhered to each other throughout the simulations. Interestingly, CB6 groups self-organize in a very regular pattern on the network by creating relatively large clusters across the surface.

As a metric that is related to porosity of our structures, we also measure the largest void-free sphere diameter of the equilibrium network structures by using the Zeo++ software^71^ (Figure 8d). This software is mainly used for the characterization of zeolite structures, and it measures the largest sphere radius (*d*_r_) that can travel through the structure in all directions. We observe that although neutral polyrotaxane network has less surface area, it has a slightly larger cavity compared to positively charged polyrotaxane-network structures. This suggest that charge-state can be used to alter the porosity of the network structure. Interestingly, the multilayer network has both larger surface area and cavities, and thus highest porosity (Figure 8d). This suggests that two layers interacting with each other further stabilize the network structure providing higher resistance to planar pressure in addition to larger voids.

## Conclusion

To summarize, by using all-atom MD simulations, we characterize the conformational and solvation properties of [5]rotaxane for various charge states, some of which can be realized at a certain ionic strengths (i.e., pH). Our simulations demonstrate that the pH level of the solution affects the molecular conformation of [5]rotaxane by altering positions of CB6 rings on their axles. This positional effect also alters the exposure of the porphyrin core of [5]rotaxane to solvent molecules. Analysis on each charge state showed that positions of the CB6 rings are mainly affected by the charge on the triazole groups. When CB6 rings are on triazole groups, the structure can fold onto itself, but if some of the CB6 rings are shuttled toward the core porphyrin or to the axle terminal, [5]rotaxane increases its size by expanding axles. Furthermore, [5]rotaxane can switch from one conformation to another by allowing at least one CB6 movement along the corresponding axle. The time scale of such conformational changes is on order of ∼1–10 ns. In addition, our simulation with polyrotaxane-network structures also confirm the possibility that [5]rotaxane can covalently assemble at liquid–liquid interfaces to form 2D films. Overall findings of our atomistic-resolution MD simulations are concordant with experimental observations and provide additional insight into the pH-based applications of light activated toxic agents or stimuli-responsive molecular switches.

## Supporting Information Available

GROMOS-GAFF force field benchmarking of final POS and NEUT states, the schematic of the polyrotaxane structure, and time traces of structural metrics (SASA, *R*_g_, *n*_HB_) for various charge states (PDF)
