# Supplementary material for: Effect of Charge State on the Equilibrium and Kinetic Properties of Mechanically Interlocked [5]Rotaxane: A Molecular Dynamics Study
Source: J Phys Chem B. 2023 Jan 30;127(5):1254–63. doi: 10.1021/acs.jpcb.2c07645 (PMC9923746; doi:10.1021/acs.jpcb.2c07645)
Supplement: Supplementary file 1 — jp2c07645_si_001.pdf [file jp2c07645_si_001.pdf]

Supplementary Information

for

Effect of Charge State on the Equilibrium and Kinetic Properties of Mechanically Interlocked [5]Rotaxane: A Molecular Dynamics Study

Ata Utku Özkan, Dönüş Tuncel, Aykut Erbaş

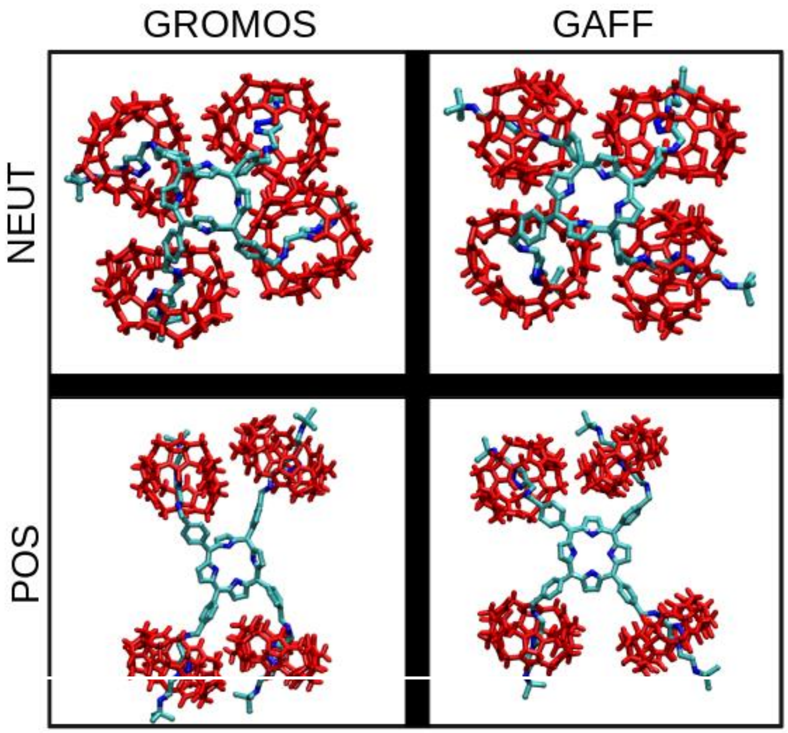

Figure S1: Comparison between GAFF – GROMOS force fields for two charge states.

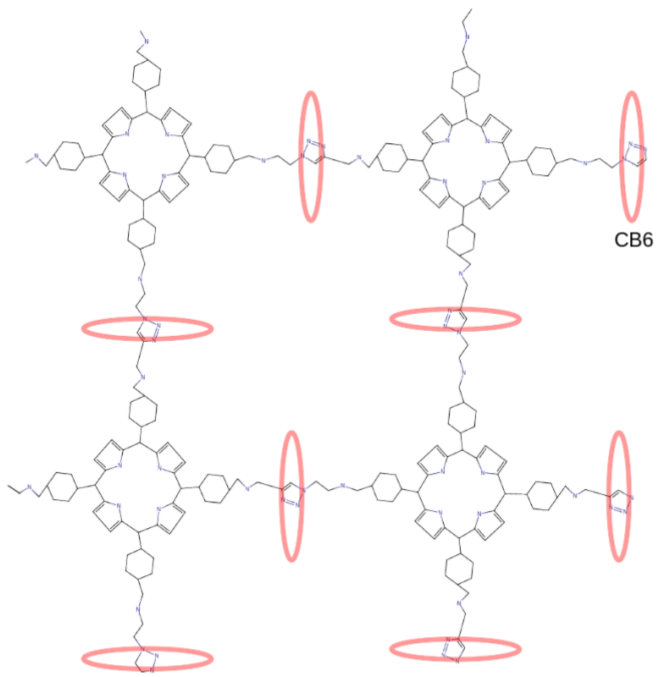

Figure S2: 2x2 Polyrotaxane Unit

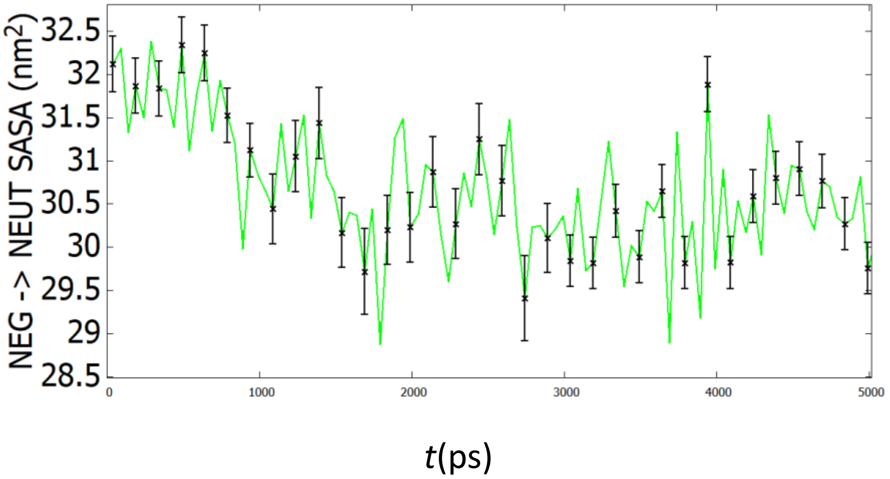

Figure S3. The solvent accessible surface area for

The NEG-to-NEUT transition.

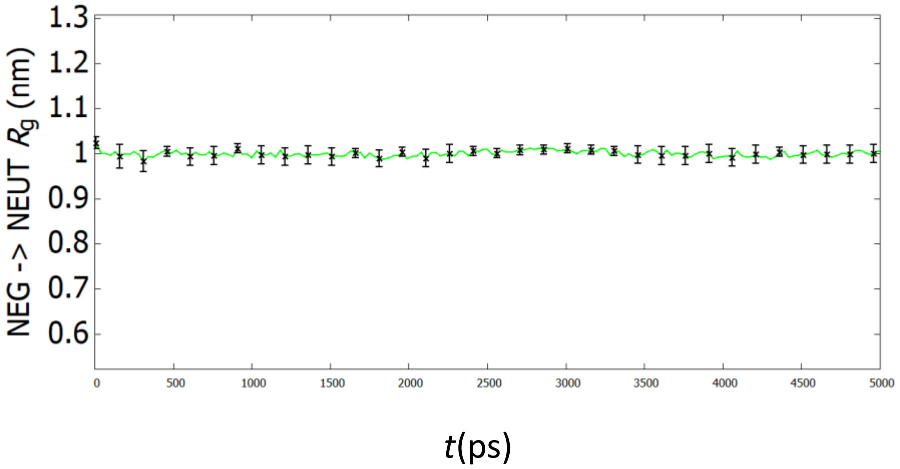

Figure S4. Radius of gyration in the NEG-to-NEUT transition.

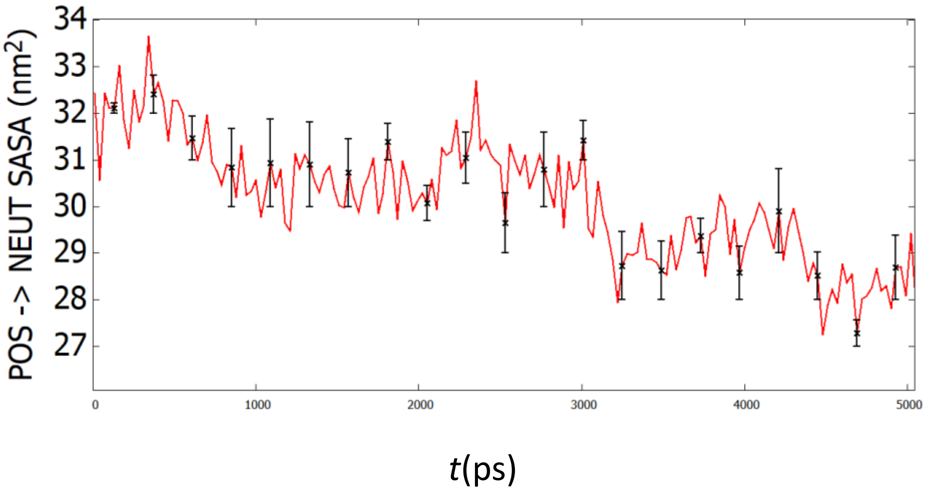

Figure S5. Solvent accessible surface area in the POS-to-NEUT transition.

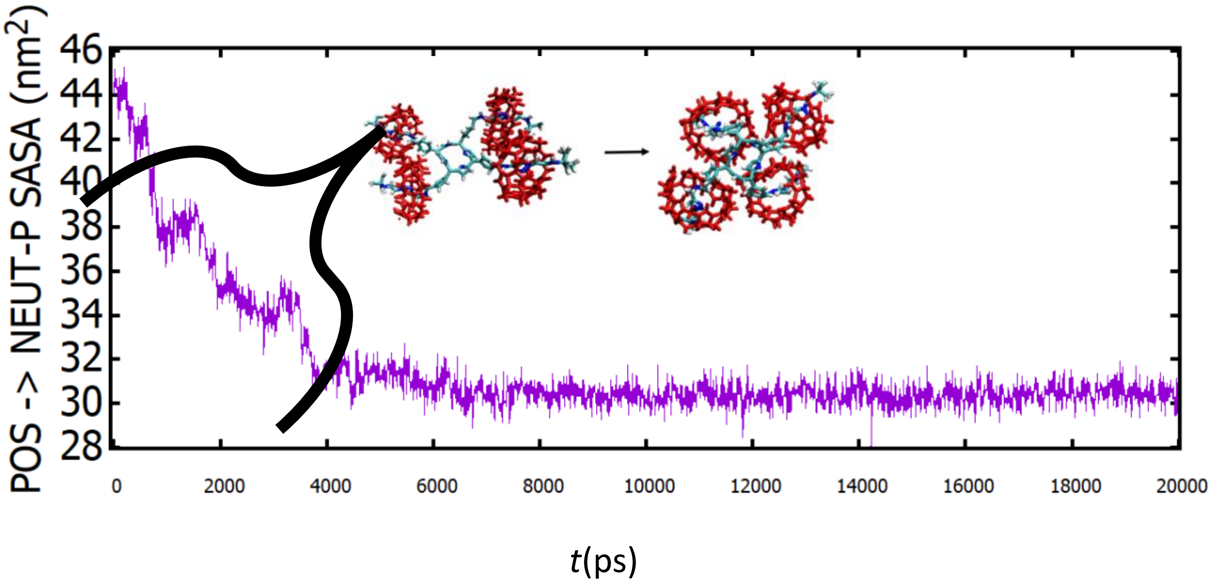

Figure S6. Solvent accessible surface area in the POS to NEUT-P transition together with a representative simulation snapshot of [5]rotaxane.

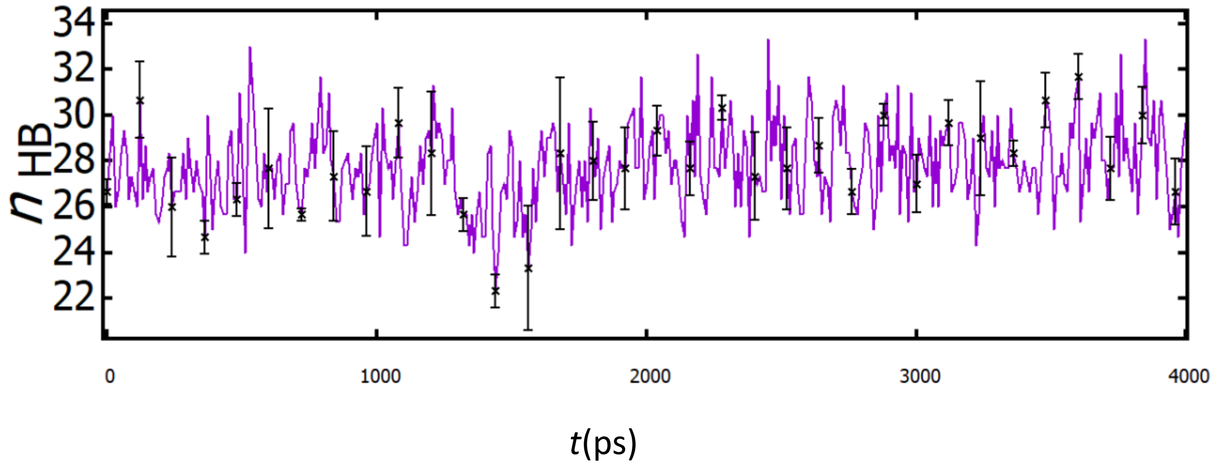

Figure S7. Hydrogen bonding between water – [5]rotaxane in the POS-to-POS-R1 transition.

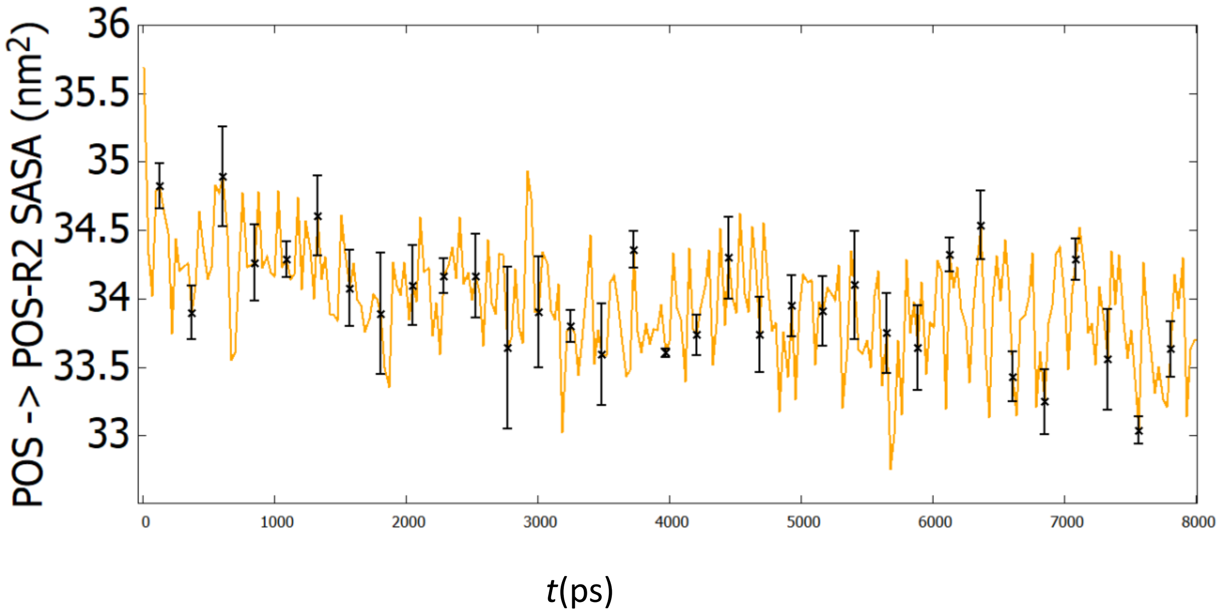

Figure S8. Solvent accessible surface area for the POS-to-POS-R2 transition.

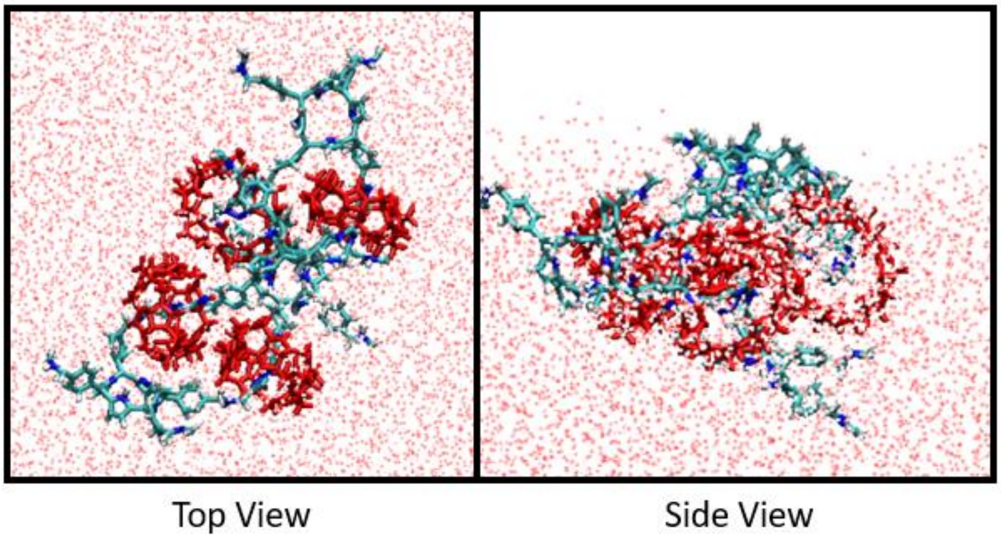

Figure S9. Single free (not a part of the periodic network) 2x2 polyrotaxane at air-water Interface.
